# Clinical and Histological Outcomes of Autologous Dentin Matrix in Post-Extraction Alveolar Healing: A Pilot Randomized Clinical Trial

**DOI:** 10.3390/jcm15020606

**Published:** 2026-01-12

**Authors:** Massiel Jáquez, Juan Algar, James Rudolph Collins, Gleny Hernández, Juan Manuel Aragoneses

**Affiliations:** 1 Department of Medicine and Medical Specialties, Faculty of Medicine and Health Sciences, Universidad Alcalá de Henares, 28801 Alcalá de Henares, Spain; massieljaquez@gmail.com; 2 Department of Periodontology, School of Dentistry, Pontificia Universidad Católica Madre y Maestra, Santo Domingo 10109, Dominican Republic; jamescollins@pucmm.edu.do; 3 University Dental Clinics, Faculty of Biomedical and Health Sciences, Universidad Europea de Madrid, 28670 Madrid, Spain; 4 Department of Dental Research, Universidad Federico Henríquez y Carvajal, Santo Domingo 10106, Dominican Republic; glenyhernandez00@gmail.com (G.H.); juanmanuel.aragoneses@euneiz.com (J.M.A.); 5 Department of Dentistry, Faculty of Health Sciences, Universidad de Euneiz, 01013 Vitoria-Gasteiz, Spain

**Keywords:** autologous dentin matrix, demineralized dentin matrix, alveolar ridge preservation, bone regeneration, BMP4 expression, extraction socket healing

## Abstract

**Background/Objectives**: Autologous dentin matrix (ADM) has been suggested as a biologically plausible biomaterial for alveolar bone regeneration after tooth extraction. However, clinical evidence regarding its biological activity and early healing outcomes is limited. This exploratory, randomized controlled pilot study aimed to descriptively assess early alveolar healing patterns and bone morphogenetic protein 4 (BMP4) expression following tooth extraction using ADM compared with other grafting approaches. **Methods**: Patients requiring tooth extraction were allocated to one of four groups: ADM, xenograft, ADM combined with platelet-rich fibrin, and a graft-free control group. Histological and immunohistochemical analyses were performed four months after extraction to descriptively assess cellular features of healing and BMP4 expression. The trial was registered at the Brazilian Registry of Clinical Trials (ReBEC; RBR-24mdgrf) and conducted under prior ethics committee approval. **Results**: BMP4 expression was detected in 67.9% of the analyzed histological fields, predominantly localized in osteocytic, osteoblastic, and medullary areas. Although descriptive differences in BMP4-positive fields were observed among the groups, no statistically significant differences were identified between the groups. Histological evaluation revealed an active cellular environment across all treatment modalities, consistent with early post-extraction healing. No adverse events related to surgical procedures or grafting materials were reported during the study period. **Conclusions**: Within the limitations of this pilot randomized clinical trial, ADM exhibited consistent biological behavior during early post-extraction alveolar healing. The observed BMP4 expression likely reflects a general physiological healing response rather than a material-specific effect. This finding supports the biological plausibility of dentin-derived grafts as osteoconductive biomaterials. These findings are hypothesis-generating, and larger, adequately powered randomized clinical trials with standardized molecular and histological assessments are required to determine their clinical relevance.

## 1. Introduction

Bone tissue regeneration is a critical aspect of both orthopedic surgery and dentistry. Following tooth extraction, alveolar bone resorption is an inevitable physiological process with relevant clinical implications. Its prevalence exceeds 90% of patients, and its extent depends on factors such as age, systemic health, and oral hygiene habits [1]. A reduction of up to 3.74 mm in horizontal width and 1.24 mm in vertical height may occur within the first six months, with more than 60% of alveolar width potentially lost during the first year [2]. One study reported a 50% reduction in crestal ridge width after one year, with the greatest drop occurring within the first 12 weeks [3,4].

Alveolar resorption predominantly affects the horizontal dimension and is more pronounced on the vestibular aspect of the alveolus [5]. Consequently, tooth extraction results not only in tooth loss but also in progressive resorption of the residual alveolar ridge. This process compromises the predictability of subsequent implant placement, increases the need for additional regenerative procedures, and may negatively affect both esthetic and functional rehabilitation outcomes [6,7].

To mitigate these changes, alveolar ridge preservation (ARP) techniques have been developed, involving immediate filling of the extraction socket with biomaterials to limit post-extraction resorption [8,9,10]. A wide range of materials has been proposed for alveolar preservation, including xenografts, allografts, and synthetic substitutes [11,12]. Among these, ADM has emerged as a biologically plausible alternative [13]. Its composition closely resembles that of bone, consisting of an inorganic phase of approximately 70–75%, mainly hydroxyapatite, and an organic phase of about 20%, of which nearly 90% is type I collagen [14]. In addition, dentin contains non-collagenous proteins such as osteocalcin, osteonectin, and phosphoproteins, as well as bone morphogenetic proteins (BMPs) capable of inducing new bone formation [14]. Among the latter, BMP4 stands out as a relevant osteogenic signaling molecule, as it regulates osteoblastic differentiation, mineralization, and tissue repair [15].

Beyond its biological similarity to bone, ADM offers several relevant clinical advantages, including immediate availability during the same surgical procedure, straightforward preparation, low cost, absence of disease transmission risk, and a minimal likelihood of eliciting an immune response [16]. BMPs play a key role in bone formation and repair [16] belonging to the transforming growth factor beta (TGF-β) superfamily [17].

Initially identified as key inducers of bone formation [18], more than 20 BMP family members have since been described with specific roles in tissue regeneration [19]. These proteins are classified into subgroups according to their structural homology, including BMP2/4, BMP5/6/7/8, BMP9/10, and BMP12/13/14 [20,21]. During embryonic development, BMPs act as morphogenic signals that regulate the migration, proliferation, and differentiation of mesenchymal stem cells into cartilage and bone tissues [22].

Molecular profiling studies have demonstrated that BMPs 2, 3, 4, 5, 6, and 7 are sequentially expressed during the bone healing process, following specific patterns [23]. Furthermore, animal models exhibiting increased expression of BMP antagonists show reduced bone quality and increased susceptibility to fractures, highlighting the role of BMP signaling not only in bone repair but also in the maintenance of adult bone homeostasis [24].

Within this family, BMP4 has emerged as a key regulator of bone and periodontal regeneration, promoting osteoblastic differentiation, mineralization, and repair of mineralized tissues [25]. Unlike BMP-2 or BMP-7, which have been more extensively studied as therapeutic agents in clinical augmentation protocols, BMP4 appears to play a regulatory role primarily during the initial stages of bone healing and tissue remodeling. It acts as an early biological signal rather than a late-stage osteogenic stimulus. Although the osteoinductive potential of ADM has been demonstrated, the specific involvement of BMP4 in alveolar regeneration processes remains incompletely understood. In this context, the assessment of BMP4 expression may provide insights into the early biological response associated with dentin-derived grafts, rather than serving as a definitive marker of clinical efficacy.

This exploratory study aimed to analyze the expression of BMP4 in ADM, which was used as a grafting material in extraction sockets. The study employed histological and immunohistochemical analyses within the framework of a pilot randomized clinical trial. The study did not intend to assess clinical efficacy or material superiority.

## 2. Materials and Methods

### 2.1. Study Design

This study was designed as a randomized, controlled pilot clinical trial conducted to exploratorily evaluate early alveolar bone healing in four experimental groups: (1) ADM; (2) xenograft; (3) autologous dentin with platelet-rich fibrin (PRF); and (4) a graft-free control group. The study involving human participants was reviewed and approved by the Ethics Committee of Federico Henríquez y Carvajal University (approval number 30/11/2020), Santo Domingo, Dominican Republic, in accordance with the Declaration of Helsinki. The trial was registered at the Brazilian Registry of Clinical Trials (ReBEC), RBR-24mdgrf (UTN: U1111-12583170). Participant recruitment took place between January 2022 and December 2022.

The study was conducted at the dentistry clinic of the Federico Henríquez y Carvajal University, Santo Domingo, Dominican Republic.

All participants were informed of the procedure and provided written informed consent and were randomly assigned (1:1:1:1) to the four study groups using a computer-generated randomization sequence created with Random.org (Random.org LLC, Dublin, Ireland; https://www.random.org, accessed on 1 January 2022). Allocation was concealed using sequentially numbered, opaque, sealed envelopes prepared by an independent researcher not involved in the clinical procedures. The surgeon opened the envelope immediately before graft placement.

Patients were blinded to the treatment group. The histological and immunohistochemical examiners were blinded to group allocation. The operating surgeon could not be blinded due to the nature of the grafting materials.

The study followed the CONSORT 2010 guidelines and the CONSORT extension for pilot and feasibility randomized clinical trials.

### 2.2. Participants

The sample size was calculated using G*Power v3.1.9.7 (Heinrich-Heine-Universität Düsseldorf). A chi-square test for contingency tables with four parallel groups was assumed, using a moderate effect size (w = 0.35), a significance level of α = 0.05, and a statistical power of 80%. The analysis indicated that a total of 48 sockets would be required to detect the prespecified intergroup differences in BMP4 expression.

However, the target sample size could not be achieved due to ethical constraints related to harvesting bone biopsies and logistical limitations inherent to the study design. Accordingly, the final sample size was reduced to 28 sockets, aligning with the exploratory purpose and methodological framework of pilot randomized clinical trials. This reduction resulted in lower statistical power than originally planned, increasing the risk of type II error and limiting the ability to detect small or moderate intergroup differences. Consequently, the study was not powered to detect definitive intergroup differences; all statistical analyses should be interpreted as exploratory and hypothesis-generating, rather than confirmatory.

Inclusion criteria were as follows: patients aged over 18, systemically healthy (American Society of Anesthesiologists (ASA) classification I), with an indication for extraction of at least one non-restorable tooth. This included cases of vertical fracture, penetrating caries, coronal fractures, or stage III–IV periodontal disease (grades B or C), as well as alveoli with a minimum residual depth of at least 3 mm.

The extractions involved anterior and posterior teeth located in the maxilla and mandible. During the randomization process, socket characteristics, including tooth type and jaw location, were not used as stratification variables. However, the random allocation process was expected to result in a balanced distribution of these characteristics across the experimental groups. Due to the exploratory design and limited sample size of this pilot randomized clinical trial, subgroup or stratified analyses based on tooth type or jaw location were not performed.

Patients who refused a bone biopsy, as well as pregnant or lactating women, individuals undergoing prolonged systemic treatment and those with trauma-altered alveolar morphology, were excluded.

Prior to the procedure, all patients underwent periodontal treatment to remove plaque and calculus and were provided with oral hygiene instructions.

A 0.2% chlorhexidine rinse was administered preoperatively, and prophylactic antibiotic therapy was prescribed after surgery (Mopen Plus containing 875 mg of amoxicillin and 125 mg of clavulanic acid, or 600 mg of clindamycin in case of penicillin allergy) [26].

### 2.3. Surgical Procedure

Following tooth extraction, all organic material, including periodontium, caries, restorations and gutta-percha, was removed. Teeth were sectioned prior to extraction when required to facilitate atraumatic removal.

The material was then processed using a Kometa-Bio Automatic Smart Dentin Grinder (Bioner, Madrid, Spain). Grinding was carried out in two cycles of three seconds each at 240 V, with a frequency of 50 Hz and at 16,000 rpm. After grinding, the resulting particles were placed in the removable compartment for nine seconds at 6 V and 10,000 rpm.

Dentin particles smaller than 1200 μm fell into the upper drawer, while those smaller than 200 μm were filtered out by the lower tray and fell into the lower drawer for subsequent extraction. The resulting material was then placed in two sterile containers, and the cleaning and disinfection processes were carried out for 10 min each, as indicated by the manufacturer.

Cone beam computed tomography (CBCT) was performed before the procedure and at four months postoperatively, coinciding with the biopsy collection.

Bone biopsies were obtained during planned surgical re-entry for implant site preparation, without additional surgical intervention for research purposes only. The biopsies were harvested using a trephine bur with an approximate diameter of 1.8 mm and a depth of 5 mm, in accordance with standard clinical protocols.

The samples were immediately fixed in formalin, individually stored, and sent to the laboratory for routine processing and paraffin embedding. Harvesting the biopsies was performed as part of routine clinical care and did not result in additional morbidity. No adverse events related to harvesting the biopsies were recorded.

A total of 28 bone samples were collected at the follow-up appointment and sent for histological and immunohistochemical analysis.

### 2.4. Histological Analysis

The 28 bone samples were decalcified in Osteomol (Merck KGaA, Darmstadt, Germany) for a standardized period of 4 h, then processed using an Autotechnicon (Bio-Hisure, Yiwu, China).

Paraffin blocks were made from these samples, and serial slices were stained with hematoxylin and eosin (H&E) for microscopic observation and histological analysis under a light microscope.

A single trained examiner performed the histological evaluation while being blinded to the treatment allocation. All samples underwent a double reading to ensure internal consistency of the observations.

Five non-overlapping microscopic fields were randomly selected and analyzed at 10× magnification for each histological section. Fields were selected at random to avoid section borders and peripheral artifacts, thereby minimizing sampling bias. The histological analysis was conducted at the level of individual microscopic fields rather than aggregated per patient or socket.

Each microscopic field contained approximately 50 cells under light microscopy. A quantitative assessment was performed in each field to determine the number of osteocytes, osteoblasts, osteoclasts, inflammatory cells (polymorphonuclear leukocytes), and vascular structures present. Other tissue components, including osteoid tissue, collagen fibers, and residual graft material, were recorded descriptively as qualitative variables, as formal morphometric quantification was not performed due to the exploratory nature of the study.

Because the analyses were performed by a single trained examiner and the study was designed as an exploratory pilot trial, formal inter- or intra-observer calibration statistics were not calculated.

### 2.5. Immunohistochemical Analysis

Three-micrometer sections were prepared and placed on Flex IHC Slides (Agilent Technology, Santa Clara, CA, USA). The samples were then placed in an oven at 60 °C for one hour.

Deparaffinization and antigen retrieval were performed using a Dako PT Link (Dako, Agilent Technology, Santa Clara, CA, USA) at 65 °C in low power mode overnight. The sample was then washed with running water and distilled water.

Endogenous peroxidase inhibition was performed using a hydrogen peroxide and methanol solution (1:9) for 1 h, after which it was washed in running water and distilled water.

The primary antibody was incubated in a Shandon-Lipshaw moisture chamber (Shandon, Cheshire, UK). The anti-BMP4 antibody (code GTX100874, Genetex, Irvine, CA, USA) was then used at a dilution of 1:200 with antibody-reducing components (Dako, Agilent Technology, Santa Clara, CA, USA) for 1 h.

It was then washed with EnVision Flex 20× Wash Buffer (Dako, Agilent Technology, Santa Clara, CA, USA). The EnVision Flex/HRP detection system (Dako, Agilent Technology, Santa Clara, CA, USA) was then placed on top for 30 min.

It was then washed again with EnVision Flex 20× Wash Buffer. The sample was developed for 15 min using a DAB kit containing chromogen and Dako Flex substrate buffer. The slide was then washed with running water and distilled water.

A copper chloride enhancer was applied for 10 min, after which it was washed under running water and distilled water. In a staining battery, it was contrasted with Mayer’s hematoxylin for 1 s and then washed under running water.

Finally, the sections were dehydrated using isopropyl alcohol, rinsed with xylene and mounted using a synthetic medium and 24 × 50 mm coverslip. The sections were observed under an optical microscope to evaluate BMP4 expression, intensity and localization.

BMP4 expression was descriptively evaluated at the microscopic field level by a single, blinded examiner. To ensure internal consistency of the observations, a double reading of all immunohistochemical sections was performed. Positivity was defined as the presence of brown chromogenic staining, as compared to a bone marrow sample that served as a positive reference control. Absence of staining was considered negative.

Staining intensity was classified using a semi-quantitative visual scale as weak (score 1), moderate (score 2), or strong (score 3) based on the visual assessment of signal intensity. The localization of BMP4 immunoreactivity was categorized as osteocytic, osteoblastic, medullary, fibroblastic, or trabecular according to the anatomical distribution of the staining.

Positive and negative controls were included in each immunohistochemical staining run, following standard laboratory protocols.

No deviations from the originally approved protocol occurred during the clinical or laboratory phases. Given the exploratory design of the study, no automated or digital image-based quantification was performed.

All primary and secondary outcomes were prespecified before data collection.

### 2.6. Statistical Analyses

Statistical analyses were performed using SPSS Statistics v24.0 (IBM Corp., Armonk, NY, USA) at a confidence level of 95% (*p* < 0.05).

The chi-square test was used to assess associations between graft material and categorical histological or immunohistochemical variables. When expected cell counts were below five, Fisher’s exact test was applied, as appropriate. Given the pilot and exploratory nature of the study, no adjustment for multiple comparisons was performed.

All quantitative outcomes were analyzed descriptively. Continuous variables derived from histomorphometric measurements were not subjected to inferential statistical testing, and one-way ANOVA was applied only in exploratory analyses when all underlying assumptions were met.

The significance level was set at *p* < 0.05.

## 3. Results

### 3.1. Study Population and Sample Distribution

A total of 36 patients were screened, and 28 of them fulfilled the eligibility criteria and were randomized. All 28 completed the 4-month follow-up and were included in the final analysis. All randomized participants contributed a single post-extraction socket to the analysis, resulting in 28 independent alveolar sites evaluated.

Group allocation was as follows: ADM (*n* = 7), ADM combined with PRF (*n* = 6), xenograft (*n* = 7), and graft-free control group (*n* = 8). There were no dropouts or protocol deviations recorded during the follow-up period.

Given the reduced sample size compared with the initial power calculation, all analyses were conducted with an exploratory intent, and the study was not powered to detect small to moderate intergroup differences.

### 3.2. Histological Findings

A histological evaluation was performed at the level of individual, non-overlapping microscopic fields. The presence of cellular and extracellular components, including osteocytes, osteoblasts, osteoclasts, inflammatory cells (PMNLs), osteoid tissue, collagen fibers, residual graft material, and vascular structures, was recorded descriptively across all experimental groups.

The chi-square test or Fisher’s exact test, depending on expected cell frequencies, was used to evaluate associations between graft material and the presence of histological components. No statistically significant associations were detected between graft material and any of the evaluated histological variables (all *p* > 0.05). Table 1 summarizes the distribution and frequency of these histological components.

**Figure 1:**
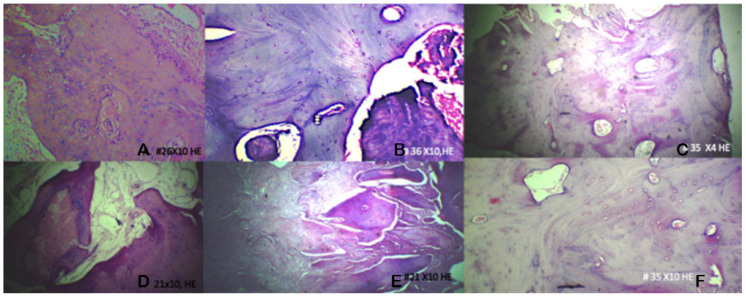
Histological sections of the regenerated alveolar bone stained with hematoxylin and eosin. (**A**) The control group 10× magnification shows representative alveolar tissue. (**B**) ADM at 10× magnification showing the newly formed bone matrix. (**C**) ADM + PRF at 10× magnification illustrating cellular components within the healing alveolar tissue. (**D**) The xenograft group at 10× magnification shows areas of osteoid material. (**E**) Presence of osteocytes within lacunae, 10× magnification. (**F**) Osteoblasts.

Representative histological features are shown in Figure 1.

### 3.3. Immunohistochemistry BMP4 Expression

**Figure 2:**
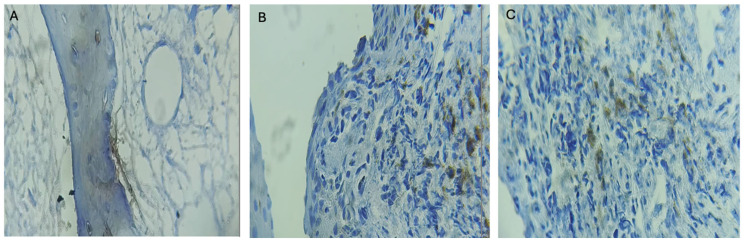
BMP4 expression according to the graft material by localization. Immunohistochemical detection of BMP-4 expression in bone tissue sections Brown arrows indicate areas of positive BMP-4 immunoreactivity (brown staining) within the bone matrix. (**A**) Control group showing minimal or absent BMP-4 expression. (**B**,**C**) Experimental groups exhibiting BMP-4 immunoreactivity in areas of bone tissue and cellular components.

BMP4 immunoreactivity was detected in the analyzed samples and observed across all experimental groups. Table 2 details the distribution of BMP4-positive and BMP4-negative cases according to graft material. Statistical analysis did not reveal a significant association between graft material and BMP4 positivity (Figure 2).

Regarding staining intensity, a majority of cases were classified as having weak BMP4 immunoreactivity, with a smaller proportion showing moderate intensity. No strong staining patterns were observed (Table 3).

Regarding localization, BMP4 expression was most frequently identified in osteocytic, medullary, and osteoblastic regions. Fibroblastic and trabecular localizations were infrequent and were therefore assessed descriptively, without inferential statistical analysis.

## 4. Discussion

Alveolar ridge preservation (ARP) is a well-established clinical strategy aimed at limiting post-extraction bone resorption and optimizing conditions for subsequent implant placement. A wide range of techniques has been described, including guided bone regeneration, socket filling with biomaterials, barrier membranes, and the use of growth factors [27,28,29,30]. Collectively, these approaches have demonstrated favorable outcomes in preserving ridge dimensions, reducing post-extraction resorption, and facilitating predictable implant-supported rehabilitation [31].

In the present pilot randomized clinical trial, histological analysis revealed no statistically significant intergroup differences among the evaluated grafting materials with regard to the presence of osteocytes, osteoblasts, osteoclasts, osteoid tissue, collagen fibers, residual graft material, inflammatory cells, or vascular components (all *p* > 0.05). Osteocytes and osteoblasts were identified in more than half of the analyzed fields, indicating the presence of an active cellular environment during the early stages of healing. This finding was consistent across all treatment modalities, and there was no evidence of material-specific effects.

Immunohistochemical analysis demonstrated BMP4 expression in 67.9% of the analyzed fields, predominantly localized in osteocytic, medullary, and osteoblastic regions. In the absence of statistically significant intergroup differences, this pattern likely reflects a general physiological healing response at the four-month time point rather than a material-specific effect. Although the ADM combined with platelet-rich fibrin (PRF) exhibited a higher proportion of BMP4-positive fields, no statistically significant intergroup differences were detected. These findings indicate that BMP4 expression occurs during the initial stages of socket healing, regardless of the grafting material used.

The localization and distribution of BMP4 expression observed in this study are consistent with previous reports describing the attributed osteoinductive potential of demineralized dentin matrices and recombinant BMP-loaded carriers [32,33,34,35,36,37,38]. However, direct comparisons should be interpreted with caution due to methodological heterogeneity, differences in healing time points, and the exploratory nature of this study.

Beyond its biological activity, ADM may offer relevant clinical advantages, including immediate availability during the same surgical procedure, straightforward preparation, low cost, absence of disease transmission risk, and avoidance of donor site morbidity associated with autologous bone harvesting [34,35,36,39,40]. In addition, osteopontin immunostaining reported in similar regenerative models supports the activation of BMP-mediated osteogenic pathways, reinforcing the hypothesis that ADM may act as a reservoir of endogenous BMP4 rather than demonstrating a direct osteoinductive effect, and may contribute to early trabecular formation [37,38].

In clinical settings, multiple reports have documented successful bone formation and alveolar ridge preservation following the use of ADM supporting its potential as a cost-effective alternative to recombinant BMP carriers [41,42,43]. Previous clinical and experimental studies have shown that ADM, which contains endogenous BMPs, may support bone regeneration by providing both an osteoconductive scaffold and a sustained release of bioactive growth factors [35,39,42]. These observations support the biological plausibility of the treatment rather than demonstrating its clinical superiority or equivalence.

Although the present pilot trial was not designed to directly compare its performance with xenogeneic grafts or recombinant BMP-2 formulations, the accumulated evidence supports the biological plausibility of ADM-mediated bone regeneration in extraction sockets [40]. These observations have been corroborated by systematic reviews and case series demonstrating the favorable results of autogenous dentin grafts for alveolar preservation and bone regeneration [44,45].

ADM may therefore be considered a biomaterial with relevant advantages over conventional alternatives. By combining osteoconductive properties with potential osteoinductive signalling, it may overcome key limitations associated with autologous bone grafts, such as donor site morbidity and surgical complexity, while avoiding the risks related to xenogenic materials [39,40,41,42,43].

In vitro studies have further demonstrated that dentin-derived particles provide a biocompatible scaffold capable of maintaining cell viability and promoting mineral deposition, supporting their regenerative potential under controlled experimental conditions [46,47].

Several clinical case reports have described higher proportions of vital bone and lower residual graft volume following the use of ADM compared with xenogenic materials. However, these findings should be interpreted with caution due to the limited sample sizes, heterogeneous methodologies, and short follow-up periods. Overall, the available evidence supports osteoconductive properties rather than definitive osteoinductive signaling [48]. In this context, the ability of ADM to act both as a structural scaffold and as a source of endogenous BMPs has been associated with enhanced mineralization and reduced ridge resorption [49,50].

Furthermore, recent clinical research has suggested that autogenous demineralized dentin matrix may yield bone regeneration results similar to those reported for xenogeneic grafts and recombinant BMP-2 carriers. However, the present pilot study was not designed to formally establish material equivalence, and larger, well-controlled clinical trials are required to confirm these observations [46,51].

In this context, a recent randomized clinical trial reported that autogenous mineralized dentin achieved comparable histomorphometric bone filling, while exhibiting a lower residual graft volume than xenografts [52]. In parallel, systematic reviews have highlighted the low incidence of adverse effects and favorable remodeling behavior associated with autogenous dentin grafts, supporting their suitability for alveolar ridge preservation [48]. Additionally, retrospective data suggest that allogeneic variants of dentin-derived matrices may offer healing outcomes comparable to those obtained with ADM, thereby expanding their potential clinical applicability [53].

Collectively, these studies support the biological plausibility and clinical feasibility of autogenous dentin-derived matrices as an alternative biomaterial for alveolar ridge preservation. Nevertheless, the available evidence is heterogeneous and is largely based on small clinical studies and short follow-up periods [44,45,46,47,48,49,50,54,55,56,57,58,59].

The inorganic phase of dentin is structurally similar to native bone, providing an osteoconductive scaffold that supports new bone formation processes. Its autologous origin significantly reduces the morbidity associated with harvesting conventional bone grafts [54,60,61]. These properties, together with its immediate availability during the same surgical procedure and its low cost, further enhance the clinical applicability of ADM in alveolar ridge preservation protocols.

Furthermore, the results of the present study do not confirm a synergistic effect between ADM and PRF. Any potential interaction must be considered speculative and hypothesis-generating, requiring further evaluation in adequately powered, controlled clinical trials. Currently, no conclusions can be drawn about additive or synergistic biological effects. Such trials are necessary to determine the clinical relevance of any interaction and define standardized application protocols.

From a practical perspective, using ADM alone or in combination with PRF may be a feasible surgical approach that could simplify clinical workflows and reduce costs [62,63]. However, assuming regenerative outcomes comparable to those of heterologous biomaterials is hypothetical and cannot be inferred from the exploratory data, which were not designed to evaluate clinical effectiveness or material equivalence

Given the exploratory design and limited sample size of this pilot randomized clinical trial, all findings should be interpreted as preliminary and hypothesis-generating. Although the prospective design and prior trial registration strengthen the internal validity of the study, the reduced number of treated sockets limits the statistical power to detect subtle intergroup differences and precludes definitive conclusions regarding material equivalence.

This study has several additional limitations that should be acknowledged. The relatively short follow-up period of four months restricts extrapolation of the findings to long-term clinical outcomes, particularly with respect to bone maturation and remodeling. Moreover, interindividual variability related to oral hygiene practices, dietary habits, and systemic conditions may have influenced the biological response, introducing a degree of heterogeneity that is difficult to fully control in a pilot clinical setting.

These limitations are consistent with those reported in previous investigations on demineralized dentin matrix, where pilot trials, case series, and studies with small sample sizes and short follow-up periods predominate [47,50,62,63,64,65,66]. Collectively, this underscores the need for larger, well-designed clinical studies with standardized protocols and extended follow-up to confirm the reproducibility and durability of the observed effects.

Future randomized, multicenter clinical trials should directly compare BMP4-mediated ADM with xenogeneic grafts and other bone substitutes, assessing not only histomorphometric outcomes but also clinically relevant parameters such as implant success, aesthetic results, and patient-reported outcomes [55,56,57]. In parallel, standardization of BMP4 quantification methods and radiographic and histological evaluation criteria will be essential to enable robust comparisons across studies and to facilitate high-quality meta-analyses [46,67,68,69,70,71,72].

Further multicenter research is also required to verify the reproducibility of ADM-mediated BMP4 expression across different patient populations and clinical scenarios [50,73]. Such prospective investigations would build upon existing evidence suggesting that autogenous demineralized dentin grafts may achieve bone regeneration outcomes comparable to those reported for xenogeneic materials and recombinant BMP-based approaches [49,74].

However, larger-scale randomized studies with longer follow-up periods are necessary to validate these findings and to establish standardized clinical protocols for ADM-mediated autologous BMP4 delivery. Long-term histomorphometric and clinical evaluations will be critical to assess the durability of the osteoinductive effect and to characterize BMP4-associated bone quality. In this context, extended follow-up studies have already reported successful ridge preservation outcomes following the use of ADM [75,76].

Randomized clinical trials have further demonstrated the safety and comparable regenerative performance of demineralized dentin matrices combined with recombinant BMPs in alveolar ridge preservation [47,61]. Accordingly, exploiting the intrinsic BMP reservoir of autogenous demineralized dentin may eliminate the need for exogenous recombinant proteins, thereby simplifying clinical protocols while preserving osteoinductive efficacy [47,63].

Conversely, emerging lines of research highlight the potential benefits of developing combinatorial strategies that combine the endogenous release of BMP4 with biodegradable scaffolds of controlled degradation, nanofibrous carriers with the sequential release of angiogenic factors and digital platforms for designing personalized scaffolds [77,78,79]. Such approaches may enhance vascularization, modulate growth factor kinetics, and improve the predictability of bone regeneration.

Ultimately, long-term clinical studies incorporating serial BMP4 assessment alongside patient-reported outcomes will be essential to validate the efficacy and safety of ADM–BMP4-based regenerative therapies [78,79]. In addition, further exploration of the immunomodulatory effects of BMP4 within the peri-implant microenvironment may contribute to improved tissue integration and long-term clinical stability [80].

## 5. Conclusions

This exploratory, randomized, clinical trial found that ADM is feasible and safe to use in post-extraction alveolar sockets. It also exhibited a consistent biological profile during early healing. Histological evaluation revealed the presence of osteocytes and osteoblasts across all treatment modalities, indicating an active early cellular environment that did not depend on the grafting material used. BMP4 expression was detected in healing alveolar sockets treated with ADM, both alone and in combination with platelet-rich fibrin. However, no statistically significant intergroup differences were observed, suggesting that BMP4 expression at four months likely reflects a general physiological healing response rather than a material-specific or synergistic effect. These findings support the biological plausibility of dentin-derived grafts as osteoconductive biomaterials without implying clinical effectiveness or equivalence. Within the limitations of this pilot study, all results should be interpreted as exploratory and hypothesis-generating. Larger, adequately powered, multicenter, randomized clinical trials with extended follow-up and standardized histological, radiographic, and molecular outcome measures, including quantitative BMP4 assessment, are required to confirm these preliminary observations and determine their clinical relevance.

## Figures and Tables

**Table 1:** Distribution of histological components according to grafting material.

**Material**	**Osteocytes**				**Inflammatory Cells (PMNL)**				**Osteoblast**				**Bone Graft Material**			
	**Absence (*n*)**	**%**	**Presence (*n*)**	**%**	**Absence (*n*)**	**%**	**Presence (*n*)**	**%**	**Absence (*n*)**	**%**	**Presence (*n*)**	**%**	**Absence (*n*)**	**%**	**Presence (*n*)**	**%**
Control	0	0.0%	8	28.6%	0	0.0%	8	28.6%	1	3.6%	7	25.0%	2	7.1%	6	21.4%
ADM	1	3.6%	6	21.4%	2	7.1%	5	17.9%	1	3.6%	6	21.4%	3	10.7%	4	14.3%
ADM + PRF	1	3.6%	5	17.9%	1	3.6%	5	17.9%	3	10.7%	3	10.7%	3	10.7%	3	10.7%
Xenograft	1	3.6%	6	21.4%	2	7.1%	5	17.9%	1	3.6%	6	21.4%	1	3.6%	6	21.4%
Total	3	10.7%	25	89.3%	5	17.9%	23	82.1%	6	21.4%	22	78.6%	9	32.1%	19	67.9%
*p*-value	0.713				0.417				0.294				0.483			
**Material**	**Osteoid Material**				**Collagen Fibres**				**Vascular Components**				**Osteoclast**			
	**Absence (** ***n*** **)**	**%**	**Presence (** ***n*** **)**	**%**	**Absence (** ***n*** **)**	**%**	**Presence (** ***n*** **)**	**%**	**Absence (** ***n*** **)**	**%**	**Presence (** ***n*** **)**	**%**	**Absence (** ***n*** **)**	**%**	**Presence (** ***n*** **)**	**%**
Control	3	10.7%	5	17.9%	3	10.7%	5	17.9%	5	17.9%	3	10.7%	4	14.3%	4	14.3%
ADM	4	14.3%	3	10.7%	3	10.7%	4	14.3%	4	14.3%	3	10.7%	6	21.4%	1	3.6%
ADM + PRF	2	7.1%	4	14.3%	4	14.3%	2	7.1%	3	10.7%	3	10.7%	4	14.3%	2	7.1%
Xenograft	2	7.1%	5	17.9%	2	7.1%	5	17.9%	2	7.1%	5	17.9%	3	10.7%	4	14.3%
Total	11	39.3%	17	60.7%	12	42.9%	16	57.1%	14	50.0%	14	50.0%	17	60.7%	11	39.3%
*p*-value	0.712				0.559				0.587				0.355			

**Table 2:** Distribution of BMP4 immunoreactivity according to grafting material.

Materials	BMP4				Total	%
	Negative	%	Positive	%		
Control	3	10.7%	5	17.9%	8	28.6%
ADM	2	7.1%	4	14.3%	6	21.4%
ADM + PRF	0	0.0%	6	21.4%	6	21.4%
Xenograft	4	14.3%	4	14.3%	8	28.6%
Total	9	32.1%	19	67.9%	28	100.0%
*p*-value	0.2487					

**Table 3:** Semi-quantitative assessment of BMP4 staining intensity according to grafting material.

Materials	Intensity				Total	%
	Weak	%	Moderate	%		
Control	5	26.3%	0	0.0%	5	26.3%
ADM	4	21.1%	0	0.0%	4	21.1%
ADM + PRF	4	21.1%	2	10.5%	6	31.6%
Xenograft	4	21.1%	0	0.0%	4	21.1%
Total	17	89.5%	2	10.5%	19	100.0%
*p*-value	0.1100					

## Data Availability

The data presented in this study are available on reasonable request from the corresponding author. The data are not publicly available due to ethical and privacy restrictions related to patient information.

## Abbreviations

The following abbreviations are used in this manuscript:

ARP	Alveolar Ridge Preservation
ADM	Autologous Dentin Matrix
DDM	Demineralized Dentin Matrix
PRF	Platelet-Rich Fibrin
BMP	Bone Morphogenetic Protein
BMP4	Bone Morphogenetic Protein 4
CBCT	Cone Beam Computed Tomography
ASA	American Society of Anesthesiologists
PMNLs	Polymorphonuclear Leukocytes
H&E	Hematoxylin and Eosin
IHC	Immunohistochemistry
TGF-β	Transforming Growth Factor Beta
